# All that is swollen and red is not infection!

**DOI:** 10.4103/0971-4065.45292

**Published:** 2008-10

**Authors:** P. George, M. S. Jhawar, B. Pawar, A. Joseph, U. George

**Affiliations:** Department of Internal Medicine, Christian Medical College and Hospital, Ludhiana, Punjab, India; 1 Department of Nephrology, Christian Medical College and Hospital, Ludhiana, Punjab, India; 2 Department of CTVS, Christian Medical College and Hospital, Ludhiana, Punjab, India; 3 Department of Radiodiagnosis, Christian Medical College and Hospital, Ludhiana, Punjab, India

**Keywords:** Cimino-Brescia fistula, surgical ligation, venous hypertension

## Abstract

Unilateral upper limb extremity swelling and pain are common presentations in clinical practice whose differential diagnoses include cellulitis, abscess, lymphoedema, and venous thrombosis. We report here the case of a renal transplant recipient with an unusual cause of upper extremity swelling and pain. His condition of native radiocephalic, arteriovenous (AV), fistula-related, venous hypertension was misdiagnosed and managed as cellulitis. This case illustrates the importance of an index of suspicion and careful clinical examination for diagnosis and thus, avoid potentially dangerous and distressing symptoms. The patient improved with a surgical AV fistula ligation.

## Introduction

Differential diagnoses of unilateral upper limb extremity swelling and pain include cellulitis, abscess, lymphoedema, and venous thrombosis. We report here the case of a patient with an unusual cause for hand swelling and pain. This case emphasizes the importance of a detailed clinical examination which was possibly ignored, resulting in misdiagnosis and potentially dangerous complications.

## Case Report

**Fig. 1:**
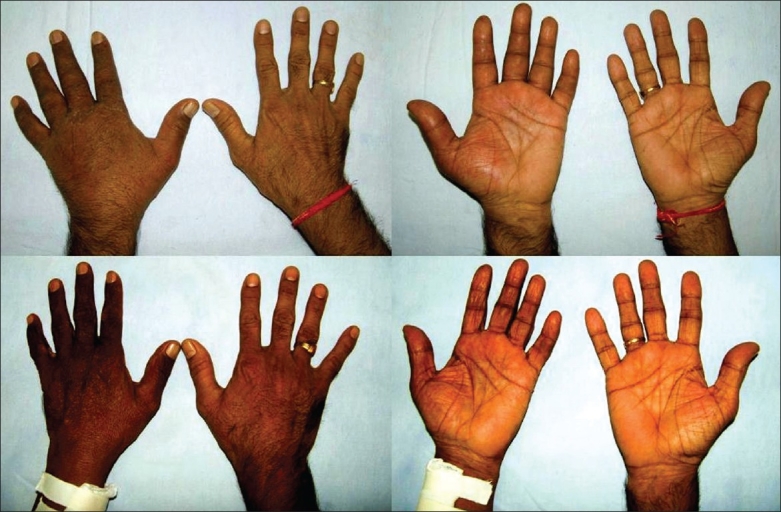
ABOVE: Swelling of the left hand with venous congestion of distal palm and fingers, BELOW: The swelling and congestion are not seen postoperatively

A 53 year-old dark-complexioned male who underwent renal allograft transplantation four years ago, presented with swelling and pain of the left hand that had progressed over the last two months. Before his admission, he was managed as a case of cellulitis with antibiotics, magnesium sulphate dressings, and antiinflammatory drugs, with no improvement of symptoms. The swelling and pain was prominent in the distal palm and fingers, and increased in the dependent position with mild improvement on limb elevation. A reddish dusky hue was noticed for a few weeks in the distal palm and fingers [Fig. 1]. There was no sensory impairment, paresthesia, or loss of movement of the hand. He denied any drug use other than his immunosupression regime of cyclosporine, azathioprine, and prednisolone. He did not smoke, consume alcohol, use illicit drugs, or operate heavy machinery.

Prior to transplantation, he was on maintenance hemodialysis using a left radiocephalic fistula for two years. Subclavian canulation or anatomical details of the fistula were unavailable as he was under treatment at another center.

Examination revealed an obvious swelling of the left hand with a minimal local rise of temperature, thinning with excoriation of the skin, and erythema of the distal palm and fingers [Fig. 1]. Tenderness of the hand, especially distal to the carpal bones was seen. There was no wasting of hand muscles and movements of the hand were restricted due to severe pain. No sensory loss or evidence of median nerve compression was present. The arterial pulsations were normal and a patent left radiocephalic fistula was observed. The veins of the forearm as well as those around the wrist were thick-walled and bulging. The dusky hue and pain were enhanced when the hand was kept in a dependent position, and mildly reduced when elevated, or when the radial artery was digitally occluded. The veins of the hand did not collapse on limb elevation.

The oxygen saturation observed by a pulse oxymeter was normal. Ultrasound Doppler imaging showed a patent radiocephalic fistula with low resistance flow in the left radial artery and increased peak systolic velocity and spectral broadening. The cephalic vein proximal to the anastomosis showed high velocity turbulent flow (139 cm/sec) and wall thickening, suggestive of arterialization with arterial pattern flow. A dilated segment distal to the elbow showed turbulent flow. The cephalic vein distal to the fistula showed no evidence of color uptake. Subclavian, axillary, and proximal cephalic vein stenosis was ruled out with a Doppler study. Angiography was not attempted due to a high risk of radiocontrast-induced nephropathy.

**Fig. 2:**
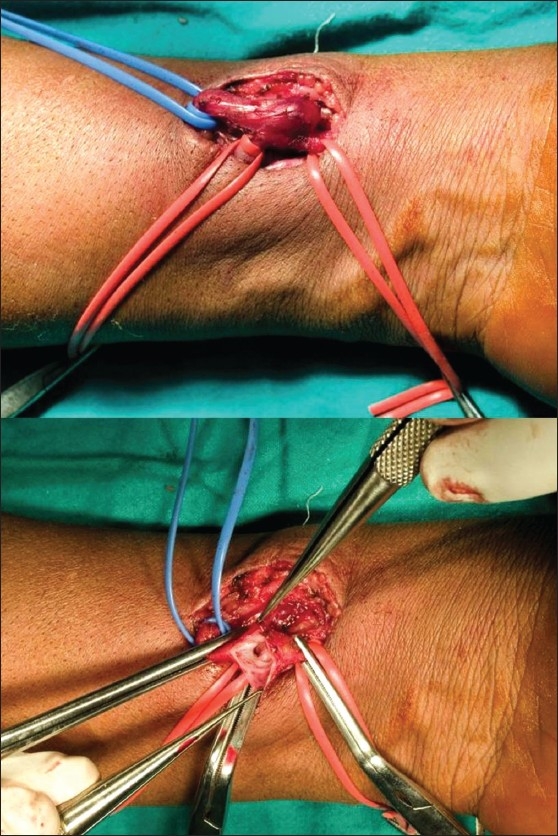
Left end-to-side arteriovenous fistula with exposed vascular anatomy prior to ligation

Venous hypertension of the left hand was diagnosed secondary to the AV fistula and revision / ligation planned. An end-to-side Cimino-Brescia fistula [Fig. 2] was seen intraoperatively with marked arterialization and dilatation of the proximal vein. Due to a difficulty in revising the fistula during surgery, it was ligated at the arterial ends; there were no complications. Within a few hours, resolution of the duskiness, pain, and swelling of the left hand was noticed postoperatively [Fig. 1].

## Discussion

A native arteriovenous (AV) fistula is the preferred access for hemodialysis^1^ and it provides adequate extracorporeal flow with reduced morbidity and mortality. Since its introduction in the early 1960s, the Cimino-Brescia fistula has been instrumental in improving dialysis and the quality of life of patients on maintenance hemodialysis. Insertion of grafts, both autologous (saphenous vein) and polymers (expanded polytetrafluoroethylene), have further revolutionized access management in patients with poor vascular anatomy.^2^ Techniques for native access include end-to-side, side-to-side, and end-to-end anastomosis of the vein and artery respectively. Venous hypertension is more commonly seen in side-to-side anastomosis due to stenosis of the central vein distal to the anastomosis, which causes reversed flow into the veins of the hand. On the other hand, only central flow is permitted in end-to-side anastomosis, thus reducing the possibility of this complication. The end-to-side anastomosis is thus preferred due to the lower incidence of venous hypertension in the hand as compared to the side-to-side procedures.^3^ Other complications commonly encountered with AV fistulas include aneurysms, arterial steal, central vein stenosis, and rarely, congestive cardiac failure.^1^

AV fistulas remain patent with no significant morbidity after renal transplantation and closure is not contemplated unless specifically indicated. Patard *et al*. reported spontaneous thrombosis of AV fistulas in 31 and 61% cases of patency on long-term follow-up. Only 7.5% of the patients required surgical closure of the AV fistula after transplantation.^4^

The hand has ten compartments: the thenar, hypothenar, adductor pollicis, three volar, and four dorsal interossei. Increased volume due to edema may present as a compartment syndrome.^5^ Peripheral venous hypertension presents with hand edema and restricted mobility. If unattended, it progresses with profound swelling of the hand and digits to venous gangrene.^6^ It can masquerade as infection and have disastrous consequences if unrecognized. Hand infection should be differentiated and should be suspected if swelling and redness of the thenar and midpalmar spaces is observed with marked swelling of the dorsum of the hand as compared to the palmar surface.^5^ Immediate treatment with antibiotics and drainage is warranted.

In this patient, peripheral venous hypertension was unfortunately unrecognized in the early stage and managed twice as cellulitis, during which time, his symptoms worsened in terms of pain, swelling, and debility. When the clinical diagnosis is not clear cut, it is useful to consider a Basal Digit Pressure (BDP) < 60 mm Hg (measured by digital photoplethysmography in the ipsilateral third finger) and Digit-to-contralateral Brachial Index (DBI) < 0.4 to be associated with hand ischemia due to AV access.^7^

Revision/ligation of the fistula removed the source of venous hypertension and resulted in immediate, postsurgical relief from pain and swelling, much to the delight of the patient.

Several notable points emerge from this case:

1. Venous hypertension of the hand is uncommon and may be missed, especially in dark-complexioned individuals. Although more common in side-to-side AV fistulas, they may also (rarely) be seen in end-to-side fistulas.
2. They may masquerade as infection leading to delayed or missed diagnosis that can be potentially dangerous.
3. A simple clinical examination with close inspection and palpation is sufficient to diagnose venous hypertension in most cases. Additionally, USG Doppler, photoplethysmography, and angiography may be useful investigations.
4. Early surgical ligation of the fistula immediately relieves all symptoms and signs of venous congestion and ischemia in limb-threatening situations.
5. Due to the fact that an end-to-side anastomosis is associated with a lower incidence of venous hypertension; a side-to-side fistula should not be used for vascular access as it offers no additional merit.

## Conclusions

Awareness and early recognition of venous hypertension as an unusual complication of native AV fistulas, particularly, side-to-side fistulas, can prevent limb-threatening complications. However, it must be emphasized from this report that possibility of venous hypertension should be considered, even in end-to-side AV fistulas.

Careful examination of the hand is all that is required for diagnosis with a surgical revision / ligation of the AV fistula to immediately relieve distressing symptoms. However, misdiagnosis or late diagnosis may result in disastrous consequences, including gangrene and amputation. The interaction of nephrologists, radiologists, and vascular surgeons is immensely beneficial in the diagnosis and management of venous hypertension arising out of AV fistula dialysis access.^1^
